# Supplementary material for: A dynamics and stability framework for avian jumping take-off
Source: R Soc Open Sci. 2018 Oct 31;5(10):181544. doi: 10.1098/rsos.181544 (PMC6227979; doi:10.1098/rsos.181544)
Supplement: Document detailing a simplified 1D mass-spring model of take-off, derivation of centre of pressure location, model sensitivity analysis, and modulation of toe-off and tipping [file rsos181544supp9.docx]

## Supplementary Material for Dynamics and Stability in Avian Jumping Take-Off

1. One-Dimensional Take-off Model Using a Linear Spring Leg

Here we consider a simple spring-mass model (Figure S1) to understand how the take-off velocity compares with the maximum velocity. By inspection, the governing dynamic equation for the body is $\ddot{y}+\frac{k}{m_{1}}y=0$, where *m_1_* is the mass of the upper body, and *k* is the stiffness of the spring connecting the two bodies. Assuming a solution for the position of the form $y=y_{o}\cos\omega t$ we also have $\dot{y}=-y_{o}\omega\sin\omega t$ for the velocity and $\ddot{y}=y_{o}\omega^{2}\cos\omega t$ for the acceleration, where $=\sqrt{\frac{k}{m_{1}}}$ . To generalise the solution, the model should be dimensionless. To achieve this we base the reference length scale on the equilibrium displacement of the body, $y_{e}=\frac{m_{1}g}{k}$, and the reference time scale on the period of oscillation, $T=\frac{2\pi}{\omega}=\frac{2\pi}{\sqrt{\frac{k}{m_{1}}}}$ . This step is not essential to the argument being made, but helps in the economy in which outputs can be presented.


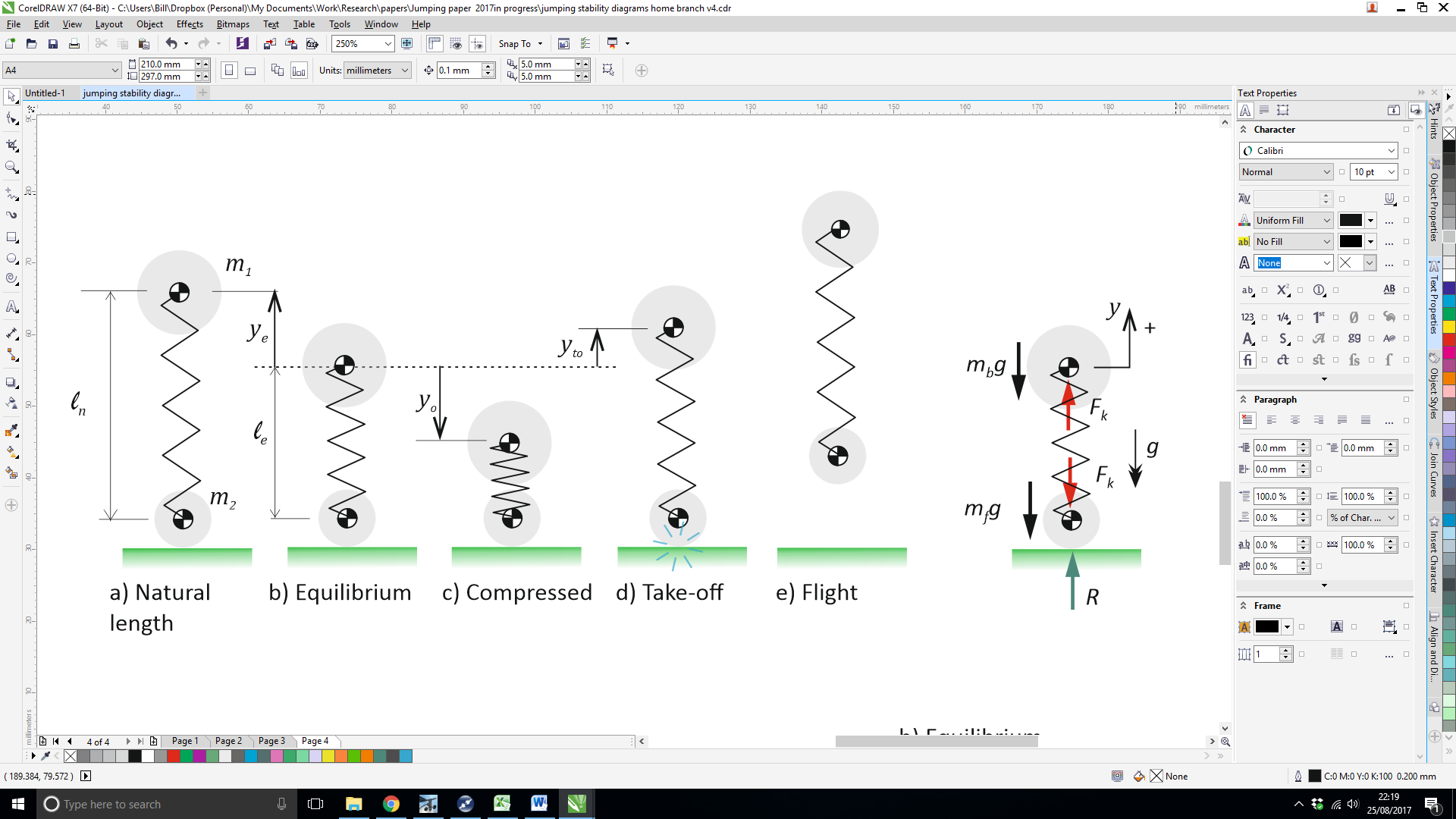


Figure S1 Single degree of freedom mass-spring jumping model. The two masses nominally represent a body and foot connected by a massless spring of stiffness *k*. The natural length $\mathcal{l}_{n}$ is the length of the spring under no load. The equilibrium length $\mathcal{l}_{e}$ is the length of the spring deflected under the static load of the body. The compressed condition corresponds to a static condition immediately prior to release. Take-off is defined as the state when the ground reaction vanishes and the foot leaves the floor. Note that the spring length will ordinarily not be the natural length at this state. Flight phase is shown for illustration only – without damping, the masses will oscillate freely and the model does not provide useful insight into this phase of the jump.

To exercise the model, consider three test cases: first, a small initial displacement for which the rate of deceleration of the body as the spring passes the equilibrium position is too small to meet the take-off criterion. Here, we expect the body to return and oscillate about the equilibrium position without the foot ever leaving the floor. Second, consider a limiting case where the initial displacement is just sufficient to meet the take-off condition but only momentarily. Finally, consider a case where the initial displacement is sufficient to precipitate a full take-off and subsequent free flight. These cases are illustrated in Figure S2.

The under actuated and critically actuated motions in Figure S2 (light and medium grey lines) are straightforward simple harmonic motion. The fully actuated case (black line) is more interesting. The filled circle marks the point where take-off occurs. Since this model has a massless foot, the critical deceleration value is -1*g* as discussed in section 2.3. The velocity graph is interesting in that it makes the point that take-off does not generally occur at the peak velocity, which a cursory intuition might expect. It does, however correspond with the spring reaching its natural extension length. This is an outcome of the fact that we are considering a massless foot (and no damping) in this analysis and is not a general result.


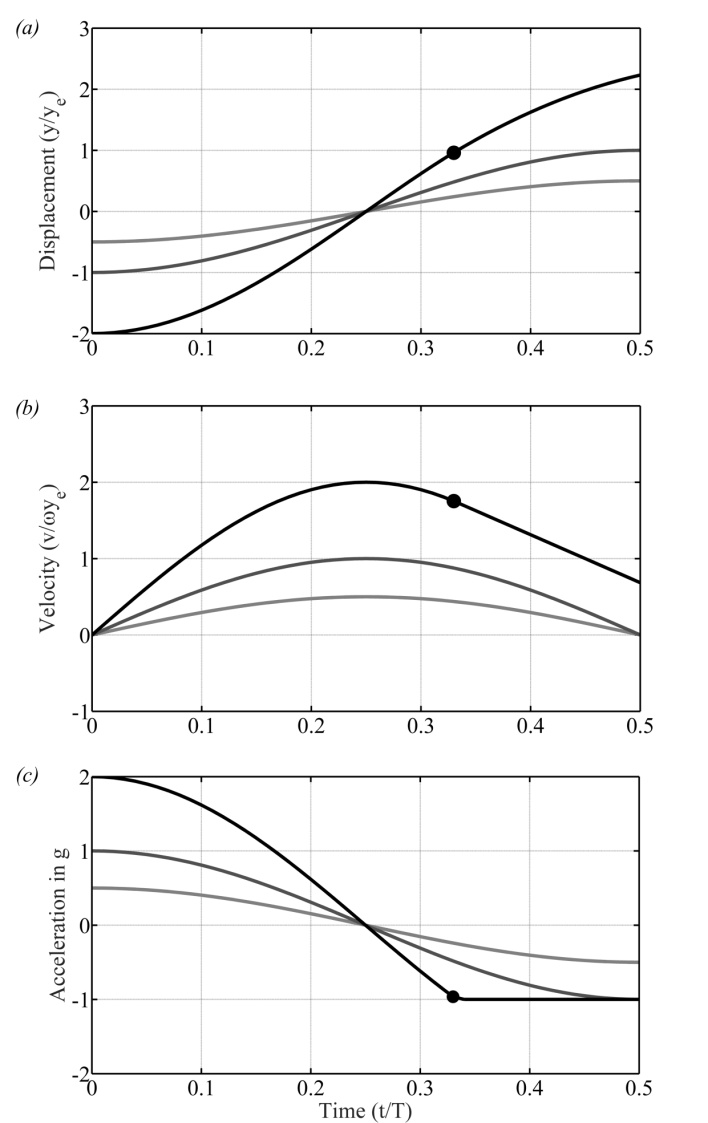


Figure S2 Dimensionless displacement, velocity and acceleration of a spring-mass jumping model for three different initial displacements. An initial displacement of -1 (medium gray line) corresponds to the critical ‘escape’ condition – for lower displacement magnitudes (light gray line) there is no take-off, greater than this (black line) and take-off does occur. An initial displacement of -1 corresponds to an initial acceleration of 1*g*. Take-off occurs when the acceleration is equal to -1*g*. Note that take-off occurs *after* the maximum velocity, i.e. the take-off velocity is less than the peak velocity. After take-off, the body continues to decelerate at –1*g*. Data in plots was generated from analytical solutions.

## 2. Derivation of centre of pressure location

In the case of zero net moment (no tipping), taking moments about the body centre of gravity (point B in Figure 4b) gives

${\mathbf{R}_{P}\boldsymbol{\times}\mathbf{r}_{\mathrm{BP}}\mathbf{-}\mathbf{T}}_{R}=0.$ E1

The net ground reaction force is comprised of inertial and gravitational loads:

$\mathbf{R}_{\mathbf{P}}=\mathbf{R}_{A}+\mathbf{R}_{C}=m{\ddot{\mathbf{r}}}_{B}-m\mathbf{g}.$ E2

Substitution of vector quantities $\mathbf{r}_{\mathrm{BP}}=[x_{B}-x_{P}, y_{B}, 0]$, ${\ddot{\mathbf{r}}}_{B}=[\ddot{x}_{B}, \ddot{y}_{B}, 0]$, $\mathbf{T}_{R}=[0, 0, T_{B}]$ (where *T_B_* is the torque applied to the body, positive anticlockwise), into equations 3 and 4, expanding the cross product, and rearranging gives the position of the CoP

$x_{P}=x_{B}-\frac{{\ddot{x}_{B}y}_{B}}{\ddot{y}_{B}-g}+\frac{T_{B}}{m\left( \ddot{y}_{B}-g \right)}$. E3

The elevation angle of the resultant force vector, termed here the 'acceleration angle', *θ*, can be defined as

$\theta=\tan^{-1} \left( \frac{\ddot{y}_{B}-g}{\ddot{x}_{B}} \right)$; E4

allowing equation E4 to be rewritten as

$x_{P}=x_{B}-\frac{y_{B}}{\tan\theta}+\frac{T_{B}}{\left\| \mathbf{R}_{P} \right\|\sin\theta}$. E5

## 3. Segmented-model sensitivity analysis to mass and inertia properties

A sensitivity analysis was conducted through multiple jumping simulation trials of the segmented-model described in section 3, using the same leg joint angle kinematics in each case. The baseline case has mass and inertia distributed on body and leg segments and a body slenderness ratio of 2 (Figure S3a). Previous experimental measurements of Guinea fowl skeletal elements were used to define the leg segment centres of mass, radii of gyration, and masses as fractions of total body mass (54, table 2). An increased inertia model assumes a body slenderness ratio of 3 (Figure S3b). A ‘massless’ leg model (Figure S3c) concentrates the total system mass of 1.42kg at the body centre of gravity; the legs were assigned a small mass value (10^-5^kg) to prevent numerical instability in the simulation.


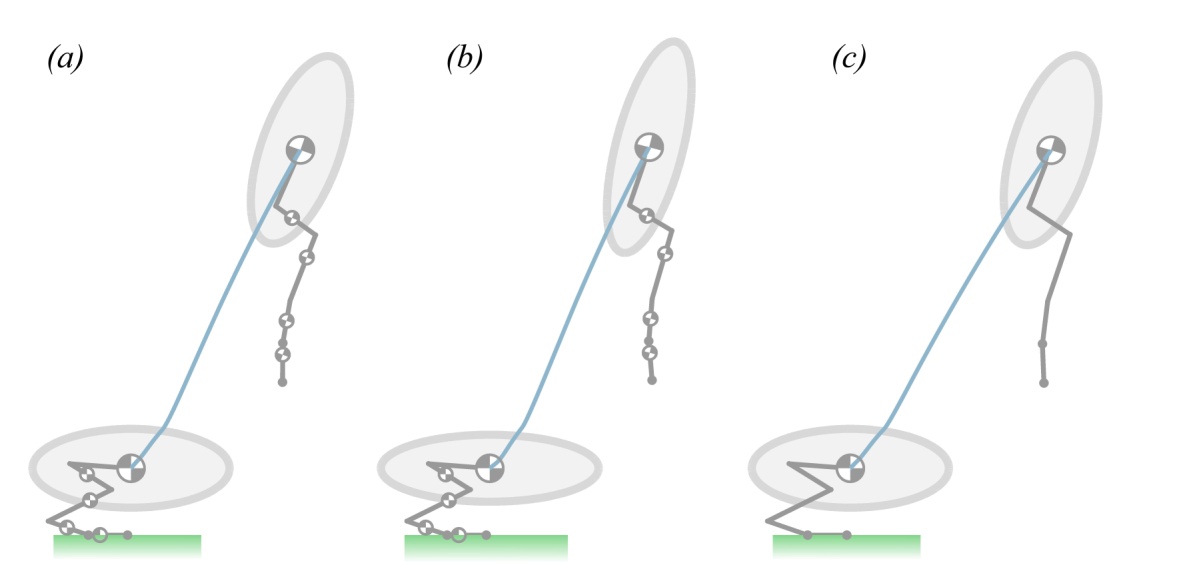


Figure S3 Jump trajectories of body centre of gravity over a time period t=0 to t=1.5*T_t-off_*. (a) Baseline, distributed mass model with a body slenderness ratio of 2. (b) Distributed mass model with increased body moment of inertia - body slenderness ratio of 3. (c) Massless leg model, with a body slenderness ratio of 2. All models have a total system mass of 1.42kg. Body ellipsoid shapes are exaggerated to show differences in body moment of inertia. Leg segment centres of gravity are illustrated smaller than body centre of gravity, but sizes are not proportional to the relative masses.

Visual inspection of the centre of gravity trajectories in Figure S3 shows the relative insensitivity of the model to the imposed changes in system inertial properties. From Figure S3a to S3b the body moment of inertia doubles, but the launch trajectory increases by only 6°, from 62° to 68° (measured from the horizontal) and launch speed increases by less than 1%. This is significant because a precise value of moment of inertia of the system is difficult to acquire experimentally. Changes of a similar magnitude occur when the system is remodelled with the ‘massless’ leg. The launch angle reduces by 5° and the launch speed by <1% going from Figure S3a to S3c. These results not only influence the modelling process but also have implications for simplifying future experimental studies that gather physical data for jumping analysis: leg segment mass and inertia are challenging to measure experimentally, particularly for small birds, yet here we show that they only have a minor influence on the jumping dynamics, so could potentially be neglected from future measurements.

## 4. Modulating toe-off and tipping

This section illustrates how the jump dynamics are influenced by strategic modification of individual joint angle kinematic time histories. We focus in particular on the dynamic features of toe-off and tipping instability. For clarity the investigation on toe-off focuses on the final loss of contact with the ground and ignores stutters, but the analysis does apply equally well to stutters.

Figure S4a shows the baseline experimental toe kinematics (2) used in the firm-ground simulations in Figures 10 and 11, along with three modified time histories. Toe kinematics are modified from the time of the minimum toe angle, with kinematics of the ankle, knee and hip remaining unchanged. A more rapid increase in toe angle causes a delay in final toe-off (blue line in Figure S4b). Increasing the toe angle more slowly than in the baseline kinematics leads to an earlier toe-off, although for the kinematics illustrated here this change is only slight and barely discernible in Figure S4b. The trends align with the theory proposed in section 2.3: a faster increase in toe-angle delays the time at which the body acceleration falls to *-g*. Figure S4b again confirms that for a massless leg system it is this acceleration condition that defines the point of toe-off. It should be noted that the modified kinematics only influence a small fraction of the jump, and for all cases in Figure S4b toe-off occurs shortly after the minimum toe angle; the steepest rise in toe angle only delays toe-off by 2% of the normalised jump time compared to the baseline case.


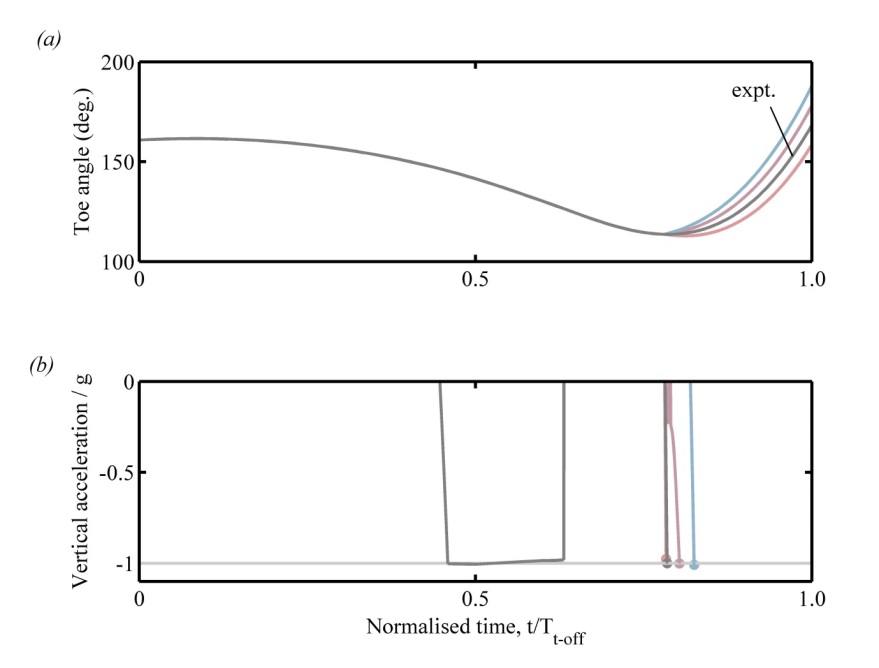


Figure S4 (a) Input kinematics for toe joint angle, defined as the angle between the functional foot and tarsometatarsus; experimental values from (2) are the dark grey line and modified values are pink, purple and blue lines. Modified angles are defined by increasing or decreasing the experimental angles linearly over time, starting at the time of minimum toe angle (*t/T_t-off_*=0.77). (b) Body vertical accelerations from simulated jumps with toe kinematics depicted in (a) with corresponding line colours; filled circles highlight toe-off times. The same experimental kinematics for hip, knee and ankle angles were used in each case (2). Light grey horizontal line represents the limiting case of vertical acceleration equal to *–g*.

If the aim of this section was to achieve a closer match between the simulated toe-off time with that observed experimentally (2) the toe angle could increase more quickly to delay toe-off even further. This approach may lead to a seemingly more accurate representation of toe-off, but would be of limited use considering other simplifications made in the model that contribute to the disparity between simulated and experimentally measured toe-off. For example, the ground compliance has already been shown to play a role in simulating toe-off. Toe-off is determined by the acceleration of the entire leg segment, so potential errors in the experimental measurements of the other joint angles are also contributors. Even errors in toe joint angle before it reaches its minimum value have an impact on the time of toe-off, as will be shown next.

Modifying the characteristic drop in toe angle is found to modulate tipping instability. The experimentally measured and modified toe angle time histories are shown in Figure S5a, and simulations using the modified kinematics are depicted in Figures S5b-d. The time *T** is an arbitrarily chosen time point used for comparison between the three cases, with *T** occurring prior to final toe-off. In all three cases at *T** the body has a positive (anticlockwise) angular velocity, but the angular acceleration differs.


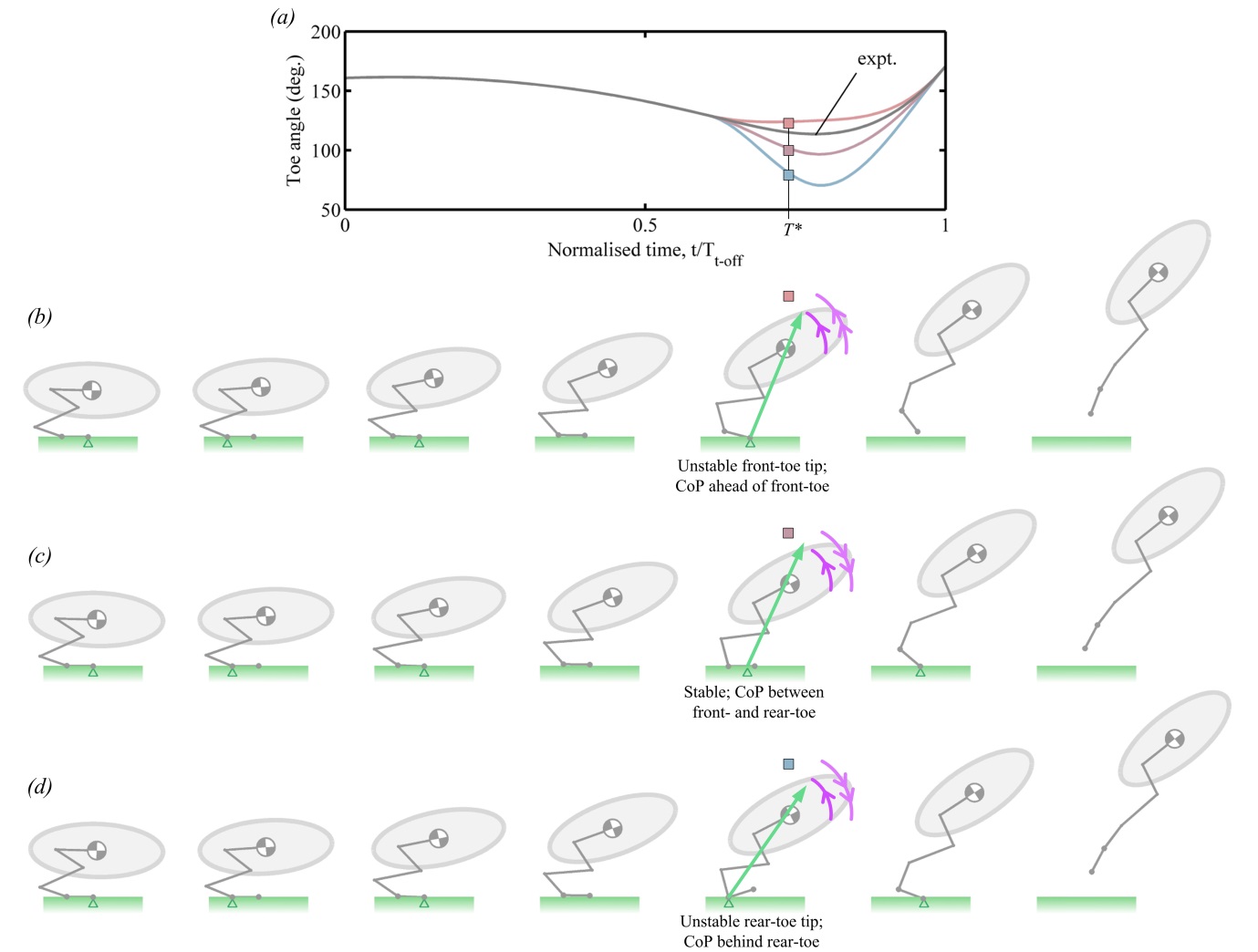


Figure S5 (a) Input toe joint angle kinematics, with experimental (grey line, (2)) and modified values (pink, purple and blue lines). Modified angles begin at the inflexion point on the curve of experimental toe angle plotted against time; the curves are defined using a cubic spline passing through three points: the first inflexion point of the experimental curve (at *t/T_t-off_* =0.6), a defined minimum toe angle at the time of the minimum experimental toe angle (*t/T_t-off_* =0.8), and a final toe angle and angular rate equal to the experimental values (at *t/T_t-off_* =1). (b)-(d) Frames from simulated jumps using experimental (b) and modified (c,d) toe joint angle kinematics; frames highlighted and labelled as unstable or stable at *T** (*t/T_t-off_* = 0.72).

At *T** the reduction in toe angle from figure S5b to S5c to S5d causes the CoP to move further aft. In Figure S5b at *T** the CoP is just forward of the front-toe and the system is unstable, with the system tipping forward (clockwise) around the front toe as the rear-toe leaves the ground. In Figure S5c at *T** the system remains stable as the CoP is between front-and rear toe, which both remain in contact with the ground. Interrogating the magnitudes of the CoP equation (equation 5) at *T** finds that from figure S5b to S5c the overriding effect is a change in the torque term, which goes from a positive to a negative value; this reduces the value of *x_p_*, moving the CoP aft of the front-toe.

In Figure S5d at *T** the CoP has moved aft of the rear-toe, causing the system to become unstable with the leg tipping backwards (anticlockwise) around the rear toe as the front-toe leaves the ground. Examining again the CoP equation at *T** we find that the vertical acceleration, $\ddot{y}_{B}$, decreases from the case in Figure S5c to S5d, causing the second term to increase and *x_p_* to decrease, moving the CoP even further aft of the rear-toe.

The body angular acceleration is positive (anticlockwise) in Figure S5b, and negative (clockwise) in Figures S5c and S5d. This is as expected considering the sign of the torque generated by the ground reaction force (green force vector) about the body centre of gravity, which corresponds to the analysis in section 2.3: in Figure S5b the ground reaction force vector passes just ahead of the centre of gravity creating a nose-up angular acceleration, and in Figures S5c and S5d is passes just behind the centre or gravity, creating a nose-down angular acceleration.

Modification of the toe-angle can be regarded as a rudimentary method of modulating the jump stability. It is not addressed here whether real animals achieve this through closed-loop control during the jump, or an open loop sequence of kinematic events. But what is evident is that for the guinea fowl there is a notable degree of robustness in the ability to achieve take-off even with the extreme ranges of instability synthesized in Figures S5b-d, which only alter the take-off trajectory by <5°. This relaxes the kinematic constraints placed upon the leg in jumping take-off, potentially making high fidelity control a less critical requirement. However, this does not consider the dynamic constraints, such as the joint torques or segment stresses, which may impose other limitations on the practicable range of leg kinematics for jumping.

5. Summary of Model Inputs

Table 1. Summary of models, input kinematics and ground contact conditions

| *Analytical models* | | | | | | |
| --- | --- | --- | --- | --- | --- | --- |
| Model Name | Figures | Videos | Leg type | No. DOF  Leg; whole system | Leg kinematics | Foot-ground contact model |
| Vertical jumping | 2, 3 | S1, S2 | Linearly extensible | 1; 2 | Constant leg acceleration | None. Foot position defined implicitly from leg kinematics. |
| Vertical spring jumping | S1, S2 |  | Linearly extensible | 1; 2 | Cosinusoidal leg acceleration | None. Foot position defined implicitly from leg kinematics. |
| 2D planar jumping | 4, 5, 6 | S3, S4, S5 | Linearly extensible + 2 revolute joints | 3; 6 | Constant leg acceleration, and joint angular acceleration | None. Foot position defined implicitly from leg kinematics. |
| *Numerical simulations; avian segmented models* | | | | | | |
| Model Name | Figures | Videos | Leg type | No. DOF  Leg; whole system | Leg kinematics | Foot-ground contact |
| Perch model  (Diamond dove) | 8, 9 | S6 | Segmented; | 4; 4 | Experimental data | Kinematically constrained |
| Ground model  (Guinea fowl) | 10, 11 | S7, S8 | Segmented | 4; 7 | Experimental data | Damped springs |
